# Beyond citation-based metrics: Measuring interdisciplinarity via SBERT semantic embeddings and its heterogeneous effects on citation impact

**DOI:** 10.1371/journal.pone.0354129

**Published:** 2026-07-22

**Authors:** Lu Liu, Yu Rong

**Affiliations:** 1 Independent Researcher, Xi’an, P.R. China; 2 Xi’an Key Laboratory for Prevention and Treatment of Common Aging Diseases, Translational and Research Centre for Prevention and Therapy of Chronic Disease, Institute of Basic and Translational Medicine, Xi’an Medical University, Xi’an, P.R. China; Max Planck Society, GERMANY

## Abstract

**Background:**

Interdisciplinary research is a cornerstone of global science policy, yet decades of research have reached conflicting conclusions about its association with citation impact. This inconsistency stems primarily from traditional indicators, which rely on reference diversity rather than genuine semantic knowledge integration, and from small, discipline-specific samples that limit generalizability.

**Objective:**

This study introduces a novel semantic interdisciplinarity measure based on Sentence-BERT (SBERT) embeddings, which directly captures cross-disciplinary knowledge integration at the textual level, and tests its heterogeneous relationship with citation impact across the full spectrum of scientific disciplines.

**Methods:**

We analyzed 121,194 articles published 2015–2025 across all 19 root-level disciplines in OpenAlex. We validated the reliability of OpenAlex disciplinary classification using multi-dimensional semantic analyses, and compared our SBERT-based indicator with the Simpson Diversity Index and Rao–Stirling Index. We employed OLS and negative binomial regressions with discipline and year fixed effects (standard errors clustered at the discipline level), journal tier heterogeneity analysis, and domain-specific decomposition analyses.

**Results:**

The semantic interdisciplinarity indicator shows moderate convergent validity with conventional citation-based metrics (r = 0.333–0.347, p < 0.001) and provides a small but meaningful increase in explanatory power beyond traditional indicators (Δ adjusted R² = 0.003), although its coefficient is marginally significant and negative (β = −1.5565, p = 0.085). Overall, semantic interdisciplinarity is positively associated with citation impact in baseline models, but this effect is primarily driven by cross-domain integration between epistemically distant domains, particularly in the natural sciences. The positive effect is consistent across all journal influence tiers, with the strongest effect observed in mid-tier journals, and presents stark heterogeneity across individual disciplines.

**Conclusion:**

Boundary-spanning research bridging epistemically distant domains appears to deliver consistent citation rewards. Our findings address long-standing inconsistencies in the literature, and provide actionable insights for research evaluation and science policy.

## Introduction

Interdisciplinary research (IDR) has become a core pillar of contemporary science policy and funding strategies worldwide. Since the seminal work of Gibbons et al. (1994) on the new mode of knowledge production, governments and academic institutions have widely embraced IDR as a key driver of scientific breakthroughs and a critical approach to addressing complex societal challenges that cannot be resolved within the boundaries of a single discipline [1,2]. This policy emphasis is rooted in a core academic consensus that integrating theories, methods, and knowledge from multiple disciplines can foster novelty and advance fundamental understanding [3]. However, whether IDR actually delivers higher scientific impact, as measured by citation performance, remains one of the most contested questions in the science of science field.

A large and growing body of literature has examined the relationship between IDR and citation impact, yet the findings are highly inconsistent and even contradictory. On one hand, a series of studies have documented a significant positive association between interdisciplinarity and citation rates, arguing that cross-disciplinary knowledge integration can expand the audience of a paper and enhance its practical and academic value [4,5]. On the other hand, an equally large number of studies have reported null or even negative effects of IDR on citation impact, suggesting that interdisciplinary work may face difficulties in gaining recognition within disciplinary communities, or may lack the depth to deliver high-quality contributions [6,7]. This long-standing inconsistency has led scholars to recognize that the relationship between IDR and impact depends critically on two core factors: how interdisciplinarity is operationalized and measured, and the heterogeneity of disciplinary combinations involved [8,9].

Traditional interdisciplinarity indicators—including the diversity of cited references [10], the Rao-Stirling index [11], and the count of distinct subject categories—have three fundamental limitations that may obscure the true IDR-impact relationship. First, these indicators are built on the untested assumption that citation patterns faithfully reflect genuine knowledge integration across disciplines. In reality, a paper may cite works from disparate fields without substantively integrating their core ideas, leading to overestimation of its interdisciplinarity [12]. Second, traditional metrics are largely insensitive to the cognitive distance between disciplines, failing to distinguish between “proximal IDR” (integrating closely related fields within the same domain) and “distal IDR” (bridging epistemically distant domains) [9]. Third, most indicators are tied to pre-defined journal or subject classification systems, which are often static and cannot capture the dynamic semantic overlap between evolving disciplines [13].

Recent advances in natural language processing (NLP), particularly pre-trained language models like Bidirectional Encoder Representations from Transformers (BERT), offer a promising solution to these limitations. Sentence-BERT (SBERT) semantic embeddings can encode the full textual content of academic papers into dense, low-dimensional vectors, capturing subtle conceptual similarities and substantive knowledge integration that are invisible to citation-based metrics [14]. By comparing a paper’s semantic embedding with the prototype vectors representing the intellectual core of different disciplines, researchers can obtain a direct, content-based measure of interdisciplinarity [15]. While this semantic approach has been applied in small-scale, domain-specific studies [16], large-scale analyses covering the full spectrum of scientific disciplines remain scarce. In particular, no study has yet used semantic embeddings to systematically decompose the heterogeneous effects of within-domain versus cross-domain IDR on citation impact across all major academic fields; existing studies are either limited to specific disciplinary domains, or rely solely on citation-based metrics, failing to capture substantive knowledge integration at the full-text semantic level across the entire scientific spectrum.

Against this backdrop, this study leverages SBERT-based semantic embeddings to construct a novel measure of interdisciplinarity, systematically validates its performance against conventional bibliometric gold standards, and examines its heterogeneous association with citation impact across the full range of scientific disciplines. We analyze a dataset of 121,194 peer-reviewed research articles published between 2015 and 2025, covering all 19 root-level disciplines in the OpenAlex database, spanning natural sciences, social sciences, and humanities. Specifically, we address three interrelated research questions:

(i) Is semantic interdisciplinarity positively associated with citation impact, after controlling for paper-level characteristics, discipline fixed effects, and publication year fixed effects?
(ii) Does the effect of semantic interdisciplinarity vary across journal influence tiers and individual disciplines?
(iii) Does the type of IDR matter? Specifically, is the citation premium of IDR driven by cross-domain interdisciplinarity between epistemically distant fields, or by within-domain interdisciplinarity between closely related disciplines?

By decomposing interdisciplinarity into within-domain and cross-domain components, and examining heterogeneity across disciplines and journal influence tiers, this study makes two core contributions to the literature. First, we develop a content-based measure of interdisciplinarity that addresses the key limitations of traditional citation-based metrics, demonstrate its incremental explanatory power beyond conventional indicators, providing a more accurate tool for evaluating interdisciplinary research. Second, we reveal that the citation payoff of IDR appears to be driven primarily by cross-domain integration between distant knowledge domains, which explains the inconsistent findings in prior literature. Our findings have direct, actionable implications for the design of research evaluation systems and science policies that aim to support genuinely integrative interdisciplinary research.

## Materials and methods

### Data source and retrieval

This study draws its data from OpenAlex [17] (https://openalex.org), an open and comprehensive scholarly graph database that indexes over 250 million scholarly with rich metadata. We focus on the 19 root‑level concepts (level‑0 disciplines) of the OpenAlex concept taxonomy, which cover the major branches of natural sciences, social sciences, and humanities.

For each discipline, we queried the OpenAlex REST API (base URL: https://api.openalex.org/works) for the period 2015–2025. The query parameters included:

- concepts.id: the concept ID of the target discipline;
- publication_year: the year range (2015-01-01|2025-12-31);
- is_paratext:false: to exclude non‑research items.

To balance representativeness and API rate limits, we adopted the following strategy: for each discipline and year, we randomly sampled up to 1,000 papers to ensure representativeness across publication years and disciplines; if a given year had fewer than 1,000 eligible papers, all were retained. Pagination was set to per-page = 200, with the page parameter incremented until either the target number of candidate papers for that year was reached or the API returned no further results. A 0.2‑second pause was enforced between consecutive requests, and a contact email was included in the request header (User-Agent) to comply with the API’s usage guidelines.

This study is based on secondary analysis of publicly available scholarly data retrieved from the OpenAlex database (https://openalex.org), which does not involve human participants, animal experiments, or collection of primary data. Therefore, ethical approval and informed consent are not applicable for this research.

### Data cleaning and sample selection

The initial sample after deduplication consisted of 122,748 papers. We then applied the following sequential cleaning steps:

- **Abstract decoding**

OpenAlex stores abstracts as inverted indexes in the abstract_inverted_index field. We reconstructed the full abstract text by parsing this field: words were placed at their corresponding index positions and concatenated in ascending order to form the full abstract text. Papers with a missing abstract or a decoded length ≤50 characters were discarded, This step reduced the sample to 121,654 papers.

- **Discipline assignment**

Each OpenAlex work is associated with a concepts list, containing concept names (display_name), association scores (score, ranging from 0 to 1), and unique identifiers. The concepts list is ordered by relevance (score descending), so we scanned this list and selected the first concept whose display_name matched one of the 19 target disciplines and had a score > 0.5. The matched discipline was then recoded into a standardized short name (e.g., “Computer science” → “computer_science”). Papers without a matching concept were excluded. No papers were excluded at this stage, leaving the sample size unchanged at 121,654 papers.

We also mapped 178 fine-grained WoS subject categories to the 19 Level-0 OpenAlex disciplines using a standardized correspondence table (S1 Table), enabling external cross-validation of disciplinary labels.

- **Author count validation**

The number of authors was extracted from the authorships field. Papers for which parsing failed or that returned an author count of zero were removed, This step further reduced the sample to 121,194 papers.

- **Construction of control variables**

**n_authors:** number of authors (already verified to be > 0);

**n_refs:** number of references (referenced_works_count, missing values filled with 0);

**is_oa:** open access status, coded as 1 if open_access[‘is_oa’] == True, otherwise 0;

**log_cite:** log‑transformed citation count, log(cited_by_count+1)

**year_cat:** publication year converted to a string, used for year fixed effects in the regressions.

The final analytical sample comprised 121,194 papers. The number of papers retained at each cleaning stage is summarized as follows: 122,748 papers after initial deduplication, 121,654 after abstract decoding, 121,654 after discipline assignment, and 121,194 after author count validation (final sample).

### Measuring interdisciplinarity with SBERT semantic embeddings

#### SBERT embedding generation.

We employed a pre‑trained Sentence Transformer model, all‑MiniLM‑L6‑v2 [18], to encode the abstract of each paper into a 384-dimensional dense vector. This model strikes an optimal balance between computational efficiency and semantic representation performance. All abstracts were processed in batches of 256, generating the final SBERT embedding matrix *embeddings_bert* with a shape of (121194, 384).

- **Disciplinary Prototype Vectors**

For each discipline, d, we computed its disciplinary prototype vector as the element-wise mean of SBERT embeddings for all papers belonging to that discipline, specified as:


$$\begin{array}{c} C_{d}=\frac{1}{N_{d}}\sum_{i\in d}e_{i} \end{array}$$

where $e_{i}$ denotes the SBERT embedding vector of paper i and $N_{d}$ is the total number of papers in discipline d**.**

- **Pairwise Interdisciplinarity Measures**

For a given paper i affiliated with discipline d, we first calculated the cosine similarity between its embedding vector and the prototype vector of every other discipline, defined as:


$$\begin{array}{c} {Sim}_{i,d^{\prime }}=\frac{e_{i}\cdot c_{d^{\prime }}}{\left\|e_{i}\right\|\;\left\|c_{d^{\prime }}\right\|}\;,\;\forall d^{\prime }\neq d \end{array}$$

These pairwise similarity scores quantify the semantic proximity between the paper’s content and the core knowledge system of each external discipline.

- **Global Interdisciplinarity Score**

We then defined the global interdisciplinarity score of paper i as the arithmetic mean of all 18 pairwise similarity scores (corresponding to the 18 external disciplines outside the paper’s home discipline):


$$\begin{array}{c} {CrossSim}_{i}=\frac{1}{18}\sum_{d^{{\;}^{\prime }}\neq d}{Sim}_{i,d^{{\;}^{\prime }}} \end{array}$$

A higher ${CrossSim}_{i}$ value indicates that the paper’s semantic content is, on average, more aligned with the knowledge cores of other disciplines, reflecting a higher degree of interdisciplinarity.

### Validation of OpenAlex Disciplinary classification

To verify the reliability and validity of the Level-0 disciplinary labels assigned by OpenAlex, we performed a multi-faceted validation procedure consisting of five complementary analytical steps:

1. **UMAP Semantic Visualization**

We reduced the high-dimensional SBERT embeddings of all sampled papers to a two-dimensional space using the Uniform Manifold Approximation and Projection (UMAP) technique, and visually inspected the clustering structure to assess the separability of the 19 disciplines.

1. **Intra- versus Inter-disciplinary Similarity Testing**

For each discipline, we computed the average cosine similarity between papers and their home-discipline prototype vector (intra-disciplinary similarity) as well as the average cosine similarity between papers and prototype vectors of all other disciplines (inter-disciplinary similarity). We then examined the statistical difference between these two types of similarity using Welch’s independent-samples t-test, which accommodates unequal variances between groups.

1. **Hierarchical Clustering of Disciplines**

Based on the pairwise cosine similarity matrix among the 19 disciplinary prototype vectors, we defined the distance metric as 1 − cosine similarity. We then performed unsupervised hierarchical clustering using Ward’s minimum variance method to identify natural, data-driven disciplinary clusters.

1. **Quantitative Clustering Evaluation**

We calculated three widely accepted clustering performance metrics to quantify the consistency between data-driven semantic clusters and OpenAlex annotated labels: clustering purity, Adjusted Rand Index (ARI), and Normalized Mutual Information (NMI).

1. **External Cross-validation with WoS Journal Categories**

Using a publicly available dataset derived from the Clarivate Master Journal List, we mapped 178 fine-grained Web of Science (WoS) journal subject categories to the 19 Level-0 disciplines in OpenAlex via a standardized correspondence rule. We then matched papers to WoS categories by journal name and computed the classification consistency rate between OpenAlex labels and WoS labels as an indicator of external validity.

### Classic interdisciplinarity indicators

To validate the performance of our proposed SBERT-based semantic interdisciplinarity indicator, we calculated two widely recognized classical interdisciplinarity metrics for benchmark comparison: the Simpson Diversity Index and the Rao-Stirling Index. Both indicators were computed based on the disciplinary distribution of cited references for each paper.

First, we retrieved disciplinary classifications for all cited references from the OpenAlex database. In total, 5,736,818 cited references were identified across all 121,194 focal papers. Of these, 5,125,768 (89.35%) were successfully retrieved from the OpenAlex API and passed basic metadata cleaning. Among the successfully retrieved and cleaned references, 3,649,607 (71.20%) were successfully mapped to one of the 19 Level-0 disciplines defined in this study. The small proportion of unmapped references primarily corresponds to books, gray literature, and very recent publications not yet fully indexed in OpenAlex. Robustness tests confirmed that excluding these unmapped references does not materially alter our core results.

1. **Simpson Diversity Index**

The Simpson index measures the disciplinary variety of references, calculated as:


$$\begin{array}{c} D=\text{1}-\sum_{i=\text{1}}^{n}p_{i}^{\text{2}} \end{array}$$

where P_i_ denotes the proportion of cited references assigned to discipline i, and n represents the total number of disciplines (n = 19). A higher value of D indicates greater disciplinary diversity in the reference list.

1. **Rao-Stirling Index**

The Rao-Stirling index integrates both disciplinary diversity and cognitive distance between disciplines, providing a comprehensive measure of interdisciplinary integration. First, we constructed a 19 × 19 inter-disciplinary distance matrix using our SBERT semantic embeddings: the distance between discipline I and discipline j (d_ij_) was defined as 1 − cosine similarity(v_i_, v_j_), where v_i_ and v_j_ are the prototype vectors of discipline i and j, respectively.

Based on this data-driven distance matrix, the Rao-Stirling index was computed as:


$$\begin{array}{c} RS=\sum_{i=\text{1}}^{n}\sum_{j=\text{1}}^{n}p_{i}p_{j}d_{ij} \end{array}$$

where p_i_ and p_j_ are the proportions of references in discipline i and j. This index quantifies the weighted average of cognitive distances between all pairs of disciplines cited by each paper, with higher values representing deeper interdisciplinary integration.

These two classical indicators were used to conduct a systematic comparison with our SBERT-based indicator, verifying its explanatory power and effectiveness in measuring interdisciplinarity.

1. **Convergent Validity and Incremental Value**

We evaluated the convergent validity of the SBERT metric by examining Pearson correlations with the two conventional indicators. We further tested its incremental explanatory power by comparing two regression models:

- **Model 1 (Baseline):** Log-transformed citation impact ~ conventional interdisciplinarity metrics + control variables (number of authors, number of references, open access status) + discipline fixed effects + year fixed effects
- **Model 2 (Augmented):** Log-transformed citation impact ~ conventional interdisciplinarity metrics + SBERT semantic interdisciplinarity metric + control variables + discipline fixed effects + year fixed effects

A statistically significant coefficient for the SBERT metric and an increase in adjusted R² in Model 2 relative to Model 1 would provide evidence that the semantic indicator captures complementary information about interdisciplinarity beyond traditional citation-based metrics.

### Statistical models

- **Baseline Regressions**

To empirically examine the relationship between interdisciplinarity and academic citation impact, we constructed two baseline regression specifications:

1. **Ordinary Least Squares (OLS)**

The dependent variable was the log‑transformed citation count log(cited_by_count+1). Control variables included: n_authors, n_refs, is_oa, as well as discipline and year fixed effects. Standard errors were clustered at the discipline level to account for intra‑disciplinary correlation.

1. **Negative Binomial Regression**

The dependent variable was the raw citation count cited_by_count. Negative binomial regression was selected over Poisson regression due to the severe overdispersion of raw citation counts (Table 1), which violates the equidispersion assumption of Poisson models. The model included the same set of control variables and fixed effects. Standard errors were again clustered at the discipline level (computed manually using statsmodels’ cov_cluster function).

**Table 1:** Descriptive characteristics of the sample. a. Descriptive statistics for continuous variables.

Variable	Mean	SD	Min	Max	N
Interdisciplinarity score (SBERT)	0.060	0.043	–0.101	0.235	121,194
Citation count	369.688	2576.193	1	801,216	121,194
Log(citation+1)	5.231	1.192	0.693	13.594	121,194
Number of authors	7.402	11.142	1	100	121,194
Number of references	92.129	112.617	0	5,000	121,194
Open access (proportion)	0.664	–	–	–	121,194

Note: The table reports mean, standard deviation (SD), minimum, maximum, and number of observations for the key continuous variables. The interdisciplinarity score is based on SBERT semantic embeddings. Citation counts are raw; the log‑transformed version is used in regression models. Open access is a binary variable; its mean represents the proportion of open‑access papers.

We used negative binomial regression as a robustness check for the baseline results, given the overdispersion of raw citation counts. All heterogeneity analyses (domain decomposition, discipline-specific, and journal tier) were conducted using OLS regression with log-transformed citations, which allows for direct interpretation of coefficients and consistent comparison across subgroups.

- **Heterogeneity Analysis**

To explore the heterogeneous effects of interdisciplinarity on citation performance across distinct disciplinary clusters, we derived three data-driven disciplinary groups based on the hierarchical clustering results of the 19 disciplinary prototype vectors. Specifically, based on the pairwise cosine similarity matrix among discipline-level prototype vectors and using Ward’s minimum variance method with distance defined as 1 − cosine similarity, the 19 disciplines were automatically partitioned into three natural clusters:

**Cluster 1: Social Sciences & Humanities:** political science, sociology, philosophy, art, history

**Cluster 2: Applied & Interdisciplinary Sciences:** geology, environmental science, geography, psychology, business, economics, chemistry

**Cluster 3: Natural Sciences & Engineering:** materials science, biology, medicine, physics, engineering, computer science, mathematics

We then estimated separate ordinary least squares (OLS) regression models for each of the three clusters, with standard errors clustered at the discipline level. All regressions used the log-transformed citation count as the dependent variable, and controlled for paper-level characteristics (number of authors, number of references, open access status), discipline fixed effects (within each domain), and publication year fixed effects.

- **Heterogeneity Analysis Across Journal Influence Tiers**

To address the potential endogeneity bias arising from journal stratification based on citation counts, and to ensure the comparability of journal influence across different academic disciplines, we partition the full sample into four mutually exclusive subgroups (Tier 1, Tier 2, Tier 3, Tier 4) using the 2025 SCImago Journal Rank (SJR) data retrieved from the official SCImago database.

Notably, these journal tiers are independently constructed within each discipline separately rather than adopting the official cross-disciplinary quartile classification. Specifically, we first merge the SJR indicator to each paper at the journal level, achieving a matching rate of 89.3%. We then rank all journals within each discipline by their SJR values and divide them into four equal-sized tiers, where Tier 1 represents the top 25% most influential journals in the discipline and Tier 4 represents the bottom 25%.

We estimate the baseline regression model separately for each self-constructed journal tier subgroup. All specifications include the full set of control variables (number of authors, number of references, and open access status), discipline fixed effects, and publication year fixed effects.

### Statistical software

All analyses were conducted in Python 3.8, using statsmodels (version 0.13.5), scikit-learn (1.2.2), numpy (1.24.3), and pandas (1.5.3).

## Results

### Sample characteristics

The final analytical sample comprised 121,194 research articles published between 2015 and 2025, covering all 19 root-level disciplines of the OpenAlex concept taxonomy. Table 1 reports the descriptive statistics for key continuous variables. The SBERT-based interdisciplinarity score had a mean of 0.060 (SD = 0.043), with a range from –0.101 to 0.235. Citation counts were highly right-skewed (mean = 369.7, SD = 2576.2, max = 801,216), consistent with the distributional characteristics of academic citation data and justifying the log-transformation in subsequent regression analyses (mean log-citations = 5.231, SD = 1.192). On average, each paper included 7.4 authors (SD = 11.1) and 92.1 references (SD = 112.6), with 66.4% of the sample being open access publications.

Table 2 presents the disciplinary composition of the sample. The largest shares came from chemistry (9.05%), materials science (9.05%), psychology (9.02%), and computer science (8.94%), while the smallest shares were from engineering (0.19%), philosophy (0.29%), and mathematics (1.48%). Table 3 shows a relatively balanced distribution of publication years, with annual counts ranging from 8,964 (2025) to 12,667 (2016). The slight decline in later years corresponds to the time lag of paper accumulation in the OpenAlex database at the time of data retrieval. The shorter citation window for recently published papers does not bias our core estimates, as we control for year fixed effects in all regression models.

**Table 2:** Distribution of papers by discipline.

Discipline	Frequency	Percentage (%)
Chemistry	10,969	9.05
Materials science	10,967	9.05
Psychology	10,928	9.02
Computer science	10,837	8.94
Biology	10,627	8.77
Environmental science	10,564	8.72
Medicine	10,557	8.71
Business	9,988	8.24
Geology	6,603	5.45
Physics	6,446	5.32
Sociology	4,189	3.46
Economics	3,827	3.16
Geography	3,539	2.92
Political science	3,047	2.51
History	2,978	2.46
Art	2,745	2.26
Mathematics	1,792	1.48
Philosophy	357	0.29
Engineering	234	0.19
Total	121,194	100

**Table 3:** Distribution of papers by publication year.

Year	Frequency	Percentage (%)
2015	12,490	10.31
2016	12,667	10.45
2017	12,098	9.98
2018	11,772	9.71
2019	11,314	9.34
2020	10,726	8.85
2021	10,655	8.79
2022	10,615	8.76
2023	10,401	8.58
2024	9,492	7.83
2025	8,964	7.4
Total	121,194	100

Table 4 reports the Pearson correlation coefficients between key variables. The interdisciplinarity score was negatively correlated with log-cited counts at the bivariate level (r=−0.220, p < 0.001). Correlations between independent variables were all below |0.22|, indicating no substantial multicollinearity risk for regression estimations.

**Table 4:** Pearson correlation coefficients among key variables.

Variable	(1)	(2)	(3)	(4)	(5)
(1) Interdisciplinarity score (SBERT)	1.000				
(2) Log(citation+1)	–0.220**	1.000			
(3) Number of authors	–0.077**	0.098**	1.000		
(4) Number of references	–0.064**	0.109**	0.080**	1.000	
(5) Open access (1 = yes)	0.108**	–0.029**	0.147**	0.073**	1.000

Note: ** p < 0.01 (two-tailed). The interdisciplinarity score is based on SBERT semantic embeddings. The sample size is N = 121,194 for all correlations.

### Semantic structure of disciplines and validation of OpenAlex classification

**Fig 1:**
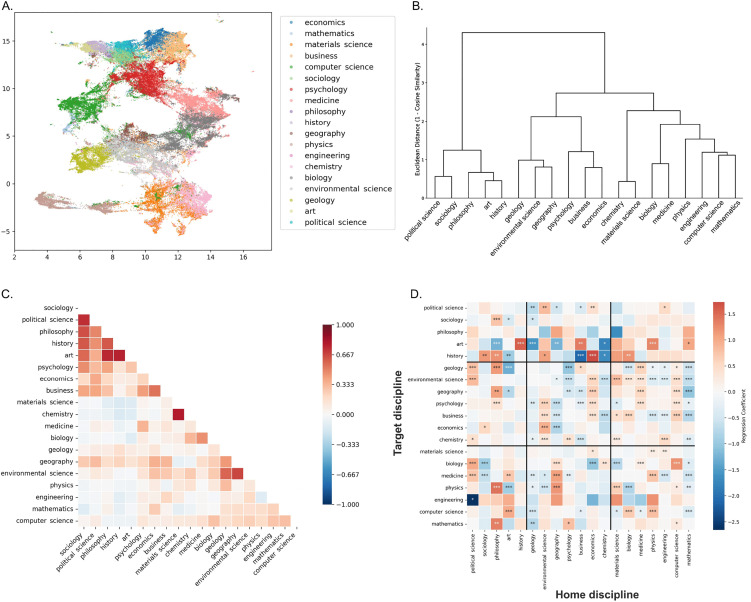
A. Two-dimensional UMAP visualization of paper-level SBERT embeddings, colored by discipline. **B:** Hierarchical clustering of disciplinary prototype vectors based on Euclidean distance (1 – cosine similarity)**. C:** Heatmap of pairwise cosine similarities between disciplinary prototype vectors. Darker red indicates higher semantic similarity, while darker blue indicates lower similarity. **D:** Heatmap of regression coefficients from discipline-specific OLS models. Rows represent the home discipline of the focal paper (citation subject: the discipline to which the paper belongs).Columns represent the target discipline (interdisciplinarity object: the discipline whose semantic similarity to the focal paper is being tested). Each cell (i, j) reports the coefficient of interdisciplinarity to the prototype vector of discipline j in predicting log-transformed citation counts for papers in discipline i, controlling for number of authors, number of references, open access status, and year fixed effects. Positive coefficients (red) indicate that higher similarity to discipline j is associated with more citations in discipline i; negative coefficients (blue) indicate the opposite. Significance is denoted by asterisks: *** p < 0.001, ** p < 0.01, * p < 0.05.

Fig 1A presents the two-dimensional UMAP projection of SBERT embeddings for all 121,194 articles, colored by their OpenAlex-assigned disciplines. The visualization demonstrates clear discipline-specific semantic clustering with well-defined boundaries between distinct academic fields. Closely related disciplines exhibit high spatial proximity, like chemistry and materials science show almost complete overlap and sociology and political science are tightly adjacent, consistent with their inherently interdisciplinary nature.

Fig 1B shows the hierarchical clustering dendrogram constructed from the pairwise cosine similarity matrix of the 19 disciplinary prototype vectors. The dendrogram reveals three major natural groupings of disciplines: (1) social sciences and humanities (political science, sociology, philosophy, art, history); (2) applied and interdisciplinary sciences (geology, environmental science, geography, psychology, business, economics, chemistry); and (3) natural sciences and engineering (materials science, biology, medicine, physics, engineering, computer science, mathematics). This data-driven grouping aligns well with conventional disciplinary taxonomies.

To quantitatively validate the reliability of OpenAlex disciplinary classification, we calculated the average within-discipline and between-discipline semantic similarities for each discipline (Table 5). For all 19 disciplines, the average within-discipline similarity was significantly higher than the average between-discipline similarity (all p < 0.001, Welch’s independent samples t-test). Within-discipline similarity ranged from 0.312 (mathematics) to 0.518 (geology), while between-discipline similarity ranged from 0.021 (engineering and mathematics) to 0.105 (geography).

**Table 5:** Descriptive statistics and clustering validity of semantic similarity across disciplines.

Discipline	N	Within similarity (Mean±SD)	Between similarity (Mean±SD)	P-value	Cluster Purity	Matching rate (%)
Art	2745	0.454 ± 0.127	0.068 ± 0.035	<0.001	0.807	0.00%
Biology	10627	0.425 ± 0.093	0.044 ± 0.031	<0.001	0.477	57.49%
Business	9988	0.440 ± 0.122	0.085 ± 0.037	<0.001	0.654	13.43%
Chemistry	10969	0.456 ± 0.123	0.033 ± 0.025	<0.001	0.441	73.64%
Computer science	10837	0.390 ± 0.174	0.050 ± 0.040	<0.001	0.528	13.77%
Economics	3827	0.470 ± 0.093	0.078 ± 0.044	<0.001	0.923	8.97%
Engineering	234	0.367 ± 0.098	0.021 ± 0.044	<0.001	0.799	87.15%
Environmental science	10564	0.469 ± 0.104	0.095 ± 0.034	<0.001	0.54	35.89%
Geography	3539	0.484 ± 0.117	0.105 ± 0.031	<0.001	0.697	7.40%
Geology	6603	0.518 ± 0.096	0.076 ± 0.027	<0.001	0.886	69.56%
History	2978	0.470 ± 0.108	0.064 ± 0.035	<0.001	0.894	30.23%
Materials science	10967	0.481 ± 0.120	0.025 ± 0.025	<0.001	0.36	14.29%
Mathematics	1792	0.312 ± 0.106	0.021 ± 0.049	<0.001	0.664	39.21%
Medicine	10557	0.397 ± 0.102	0.035 ± 0.035	<0.001	0.706	87.39%
Philosophy	357	0.465 ± 0.108	0.095 ± 0.033	<0.001	0.549	0.00%
Physics	6446	0.445 ± 0.158	0.025 ± 0.041	<0.001	0.554	83.22%
Political science	3047	0.454 ± 0.110	0.093 ± 0.036	<0.001	0.827	0.00%
Psychology	10928	0.420 ± 0.093	0.080 ± 0.031	<0.001	0.599	18.72%
Sociology	4189	0.500 ± 0.086	0.102 ± 0.033	<0.001	0.824	1.66%

**Note:** Within similarity denotes the average semantic similarity of papers within the same discipline. Between similarity refers to the average semantic similarity between papers of different disciplines. P-value reports the significance level of the mean difference between within and between similarity. Cluster purity measures the internal coherence of hierarchical clustering results. Matching rate represents the consistency between original discipline classification and algorithm-based clustering assignment.

We further assessed the consistency between paper-level semantic clusters and OpenAlex annotated labels using three standard clustering performance metrics: cluster purity, Adjusted Rand Index (ARI), and Normalized Mutual Information (NMI). The average cluster purity across all disciplines was 0.664, with particularly high purity observed for economics (0.923), geology (0.886), and history (0.894). External cross-validation using WoS journal categories yielded an average matching rate of 35.89%, with the highest rates for medicine (87.39%) and engineering (87.15%).

### Convergent validity and incremental explanatory power

We evaluated the convergent validity of the SBERT-based semantic interdisciplinarity indicator by comparing it with two widely used conventional citation-based interdisciplinarity metrics: the Simpson Diversity Index and the Rao-Stirling Index. Table 6 shows the pairwise Pearson correlation coefficients between these indicators. The SBERT indicator exhibited moderate positive correlations with both the Simpson Diversity Index (r = 0.333, p < 0.001) and the Rao-Stirling Index (r = 0.347, p < 0.001), indicating acceptable convergent validity. The very high correlation between the two conventional indicators (r = 0.921, p < 0.001) suggests substantial overlap in the information they capture about interdisciplinarity.

**Table 6:** Convergent validity and incremental explanatory power of the semantic interdisciplinarity indicator compared with conventional bibliometric metrics. a. Correlation Matrix.

Variables	Cross sim	Simpson div	Rao stirling
Cross sim	1		
Simpson Diversity	0.333***	1	
Rao-Stirling	0.347***	0.921***	1

**Note:** Pairwise Pearson correlation coefficients among the semantic interdisciplinarity indicator (cross sim), Simpson Diversity Index, and Rao–Stirling Index. *** p < 0.001.

To test whether the SBERT indicator provides incremental explanatory power beyond traditional metrics in predicting citation impact, we estimated nested OLS regression models (Table 7). Model 1, which included only the two conventional indicators and control variables, yielded an adjusted R² of 0.506. Model 2, which added the SBERT semantic interdisciplinarity indicator, showed a marginal numerical increase in adjusted R² to 0.509 (ΔR² = 0.003). Notably, the coefficient for the SBERT indicator changed from positive and significant in the baseline model (β = 0.5564, p < 0.01) to negative and marginally significant in the augmented model (β = −1.5565, p = 0.085).

**Table 7:** Incremental explanatory power of the semantic interdisciplinarity indicator in predicting citation impact.

Variables	Model 1 (Traditional)	Model 2 (Traditional + SBERT)
Simpson Diversity Index	Included	Included
Rao-Stirling Index	Included	Included
SBERT semantic interdisciplinarity	—	−1.5565 (p = 0.085)
Control variables (n_authors, n_refs, is_oa)	Included	Included
Discipline fixed effects	Included	Included
Year fixed effects	Included	Included
Adjusted R²	0.506	0.509
ΔR²	—	0.003

**Note:** OLS regression results with log-transformed citation count as the dependent variable. Model 1 includes only conventional interdisciplinarity indicators (Simpson Diversity and Rao–Stirling) and control variables. Model 2 adds the semantic interdisciplinarity indicator (cross sim). Controls include number of authors, number of references, open access status, and publication year. Robust standard errors are clustered at the discipline level. cross_sim coefficient: β = −1.5565, p = 0.085.ΔR² denotes the change in adjusted R² between Model 1 and Model 2.

### Disciplinary semantic similarity

We calculated the cosine similarity between the SBERT-based prototype vectors of the 19 root-level disciplines to map their semantic relationships, with the full similarity matrix presented in Fig 1C.

The results show distinct disciplinary clustering patterns. First, natural science and engineering disciplines formed a tightly interconnected cluster. The highest pairwise similarity was observed between chemistry and materials science (r = 0.793). Strong semantic links were also found between geology and environmental science (r = 0.585), as well as between biology and medicine (r = 0.460). Second, social sciences and humanities disciplines constituted a separate cohesive cluster, with the highest pairwise similarity between art and history (r = 0.768), followed by art and philosophy (r = 0.757) and sociology and political science (r = 0.740).

Several disciplines occupied cross-domain intermediate positions. Psychology showed moderate similarity to both sociology (r = 0.464) and medicine (r = 0.338); economics and business were closely linked (r = 0.547) and had substantial correlations with political science and sociology; geography had strong correlations with both environmental science (r = 0.667) and political science (r = 0.310). The lowest pairwise similarities were observed between art and chemistry (r=−0.119), as well as between history and physics (r=−0.032).

### Baseline regression results

Table 8 reports the baseline regression results of the SBERT-based interdisciplinarity score on citation impact. Both the OLS and negative binomial models included discipline and year fixed effects, with standard errors clustered at the discipline level.

**Table 8:** Baseline regression results of interdisciplinarity on citation impact.

Variables	(1) OLS	(2) Negative binomial
Interdisciplinarity score (SBERT)	0.5564**	1.4408**
	(0.1767)	(0.4546)
Number of authors	0.0043**	0.0058**
	(0.0003)	(0.0011)
Number of references	0.0005**	0.0004*
	(0.0001)	(0.0002)
Open access (1 = yes)	−0.0135	0.0113
	(0.0127)	(0.0408)
Constant	3.4250**	3.8128**
	(0.0222)	(0.0416)
Fixed effects		
Discipline FE	Yes	Yes
Year FE	Yes	Yes
Observations	121,194	121,194
(Adjusted) R²/ Pseudo R²	0.798	0.087

**Note:** ** p < 0.01, * p < 0.05. Robust standard errors clustered at the discipline level are reported in parentheses. The OLS model uses the log‑transformed citation count as the dependent variable; the negative binomial model uses the raw citation count, with coefficients reflecting changes in the log count. Discipline and year fixed effects are included but not shown (available in the Appendix).

The interdisciplinarity score had a positive and statistically significant association with citation impact in both specifications. In the OLS model with log-transformed citations as the dependent variable (Column 1), the coefficient of the interdisciplinarity score was 0.5564 (p < 0.01). In the negative binomial model with raw citation counts as the dependent variable (Column 2), the coefficient was 1.4408 (p < 0.01), consistent with the baseline OLS results.

For control variables, the number of authors and number of references had positive and statistically significant coefficients in both models, while the coefficient of open access status was not statistically significant (p > 0.1) in either specification. The null effect of open access status may be explained by the fact that discipline and year fixed effects absorb most of the variation in open access penetration across fields and time. The OLS model had an R² of 0.798, and the pseudo-R² of the negative binomial model was 0.087, which is within the expected range for count-data regressions with large sample sizes.

### Discipline-specific heterogeneity

We estimated separate OLS regressions for each of the 19 root-level disciplines to examine the heterogeneous effects of interdisciplinarity across fields, with year fixed effects and full control variables included in all specifications. Table 9 reports the coefficient, cluster-robust standard error, p-value, and 95% confidence interval of the interdisciplinarity score for each discipline.

**Table 9:** Discipline‑specific regression results.

Discipline	N	Coefficient	Cluster SE	p-value	95% CI
Mathematics	1,792	2.325	0.354	<0.001	[1.631, 3.019]
History	2,978	2.122	0.499	<0.001	[1.144, 3.100]
Biology	10,627	1.423	0.171	<0.001	[1.089, 1.757]
Computer science	10,837	1.134	0.152	<0.001	[0.836, 1.431]
Psychology	10,928	1.028	0.147	<0.001	[0.740, 1.315]
Philosophy	357	1.02	1.098	0.353	[-1.132, 3.172]
Chemistry	10,969	0.938	0.165	<0.001	[0.616, 1.261]
Sociology	4,189	0.662	0.23	0.004	[0.210, 1.113]
Environmental science	10,564	0.409	0.16	0.01	[0.096, 0.722]
Political science	3,047	0.206	0.281	0.464	[-0.345, 0.758]
Medicine	10,557	0.194	0.162	0.232	[-0.124, 0.511]
Geography	3,539	0.174	0.257	0.497	[-0.329, 0.678]
Materials science	10,967	0.134	0.147	0.36	[-0.153, 0.422]
Business	9,988	0.09	0.136	0.511	[-0.177, 0.356]
Physics	6,446	–0.026	0.166	0.873	[-0.352, 0.299]
Economics	3,827	–0.084	0.202	0.678	[-0.479, 0.312]
Engineering	234	–0.103	0.667	0.878	[-1.410, 1.204]
Geology	6,603	–0.478	0.207	0.021	[-0.884, –0.072]
Art	2,745	–1.554	0.525	0.003	[-2.583, –0.525]

**Note:** The table reports results from discipline‑specific OLS regressions with log‑transformed citation count as the dependent variable. Each row corresponds to a separate regression estimated using only papers from that discipline. All models include year fixed effects and control for number of authors, number of references, and open access status. Standard errors are clustered at the discipline level (within each discipline, clustering is irrelevant but consistent with the main approach). The 95% confidence intervals are based on the cluster‑robust standard errors.

The results show substantial cross-disciplinary heterogeneity. A positive and statistically significant association between interdisciplinarity and citation impact was found in 8 disciplines: mathematics, history, biology, computer science, psychology, chemistry, sociology, and environmental science. The largest coefficients were observed in mathematics (β = 2.325, p < 0.001), history (β = 2.122, p < 0.001), and biology (β = 1.423, p < 0.001). Conversely, a significant negative association was detected in art (β=−1.554, p = 0.003) and geology (β=−0.478, p = 0.021). For the remaining 9 disciplines (philosophy, political science, medicine, geography, materials science, business, physics, economics, and engineering), the coefficient of the interdisciplinarity score was not statistically significant (p > 0.05).

### Domain-level decomposition analysis

Table 10 presents the decomposition results of semantic interdisciplinarity effects across the three broad scientific domains. The positive association between semantic interdisciplinarity and citation impact was primarily driven by between-domain interdisciplinarity in the Natural Sciences domain (β = 0.364, p = 0.017), while within-domain interdisciplinarity showed no significant effect (β = 0.109, p = 0.866). In the Applied & Interdisciplinary Sciences domain, both within-domain (β = 0.195, p = 0.060) and between-domain interdisciplinarity (β = 0.443, p = 0.091) showed marginally significant positive associations. No significant associations were observed for the Social Sciences & Humanities domain. These results support a consistent cross-domain asymmetry: interdisciplinarity bridging epistemically distant domains yields stronger citation benefits than integration of closely related disciplines within the same domain.

**Table 10:** Effects of within- and between-cluster interdisciplinarity on citation impact by subsample.

Subsample	Type of interdisciplinarity	Coefficient	Cluster robust SE	p value	95% CI	N
Social Sciences & Humanities	Within-cluster	−0.0613	0.2825	0.8281	[-0.6149, 0.4923]	13316
	Between-cluster	0.6525	0.411	0.1124	[-0.1531, 1.4581]	13316
	Overall interdisciplinarity	0.646	0.411	0.1649	[-0.1531, 1.4581]	13316
Applied Sciences	Within-cluster	0.1953	0.1037	0.0596	[-0.0079, 0.3985]	56418
	Between-cluster	0.4426	0.2622	0.0914	[-0.0713, 0.9566]	56418
	Overall interdisciplinarity	0.3561	0.2622	0.1499	[-0.0713, 0.9566]	56418
Natural Sciences	Within-cluster	0.1094	0.6463	0.8656	[-1.1573, 1.3761]	51460
	Between-cluster	0.3643	0.152	0.0166	[0.0663, 0.6623]	51460
	Overall interdisciplinarity	0.3573	0.152	0.0756	[0.0663, 0.6623]	51460

**Note:** The dependent variable is log-transformed citation count. Within-domain interdisciplinarity refers to the average semantic similarity between a paper and the prototype vectors of other disciplines within the same broad cluster. Between-domain interdisciplinarity denotes the average semantic similarity to disciplines belonging to the other two broad clusters. All specifications control for number of authors, number of references, open access status, as well as discipline and year fixed effects. Standard errors are clustered at the discipline level.

### Pairwise disciplinary effect analysis

We further estimated directional discipline-specific regressions where log-transformed citation counts were regressed on pairwise semantic interdisciplinarity scores with each of the other 18 disciplines, controlling for paper-level covariates and year fixed effects. Fig 1D presents the results as a directional heatmap: rows correspond to the home discipline of the focal paper, and columns correspond to the target discipline whose semantic similarity is being evaluated. Each cell (i,j) quantifies how the semantic alignment of a paper from discipline i to the intellectual core of discipline j affects its citation impact. The results support a robust cross-domain asymmetry: statistically significant positive citation effects were almost exclusively observed in cross-domain disciplinary pairs, while within-domain pairs were predominantly non-significant or significantly negative.

### Heterogeneous Effects Across Journal Influence Tiers

We examined the heterogeneous effects of semantic interdisciplinarity across journal influence tiers by partitioning journals into four equal-sized groups within each discipline based on 2025 SCImago Journal Rank (SJR) values (Table 11). Semantic interdisciplinarity showed a statistically significant positive association with citation impact across all four journal tiers (all p < 0.001). The strongest effect was observed in mid-tier journals (Tier 3: β = 0.925), followed by bottom-tier journals (Tier 4: β = 0.747), second-tier journals (Tier 2: β = 0.733), and top-tier journals (Tier 1: β = 0.662).

**Table 11:** Heterogeneous effects of semantic interdisciplinarity across journal influence tiers.

Journal Tier	Coefficient	Cluster SE	p value	95% CI	N
Tier1	0.6624	0.1414	<0.001	[0.3852, 0.9396]	27695
Tier2	0.7331	0.1608	<0.001	[0.4180, 1.0483]	30134
Tier3	0.9246	0.139	<0.001	[0.6521, 1.1970]	26489
Tier4	0.7465	0.2079	<0.001	[0.3391, 1.1538]	23934

**Note:** This table reports how the citation benefits of cross-disciplinary interdisciplinarity vary across four journal influence tiers. Tiers are constructed by ranking journals within each discipline by their 2025 SCImago Journal Rank (SJR) values and dividing them into four equal-sized groups: Tier 1 (top 25% most influential journals in the discipline), Tier 2 (25%−50%), Tier 3 (50%−75%), and Tier 4 (bottom 25%). The dependent variable is log-transformed citation count. All specifications control for number of authors, number of references, open access status, as well as discipline and year fixed effects. Standard errors are clustered at the discipline level.

## Discussion

This study investigates the relationship between interdisciplinary research and citation impact using a novel SBERT-based semantic embedding approach. Based on 121,194 articles across all 19 root-level OpenAlex disciplines (2015–2025), our analyses suggest a statistically significant positive relationship between semantic interdisciplinarity and citation impact. Importantly, this relationship appears to be highly heterogeneous: it varies considerably across disciplines, appears to be driven mainly by cross-domain integration, and shows consistent positive associations across all journal influence tiers, with the most pronounced beneficial effects observed in mid-tier journals. These findings may help resolve long-standing inconsistencies in the literature via our novel semantic measurement approach, and advance understanding of interdisciplinarity and research evaluation in key, evidence-based ways.

We first performed a multi-stage validation framework to verify the structural validity of our SBERT‑based semantic representations and the reliability of OpenAlex disciplinary classification, followed by direct comparisons with two conventional citation‑based interdisciplinarity metrics (the Simpson Diversity Index and the Rao–Stirling Index). For structural validation, UMAP visualization and hierarchical clustering of disciplinary prototype vectors revealed clear and well‑separated semantic groupings, with closely related disciplines forming compact clusters. We then compared within‑discipline and between‑discipline semantic similarity: for all 19 disciplines, average within‑discipline similarity was significantly higher than between‑discipline similarity (all p < 0.001), supporting the semantic coherence of disciplinary boundaries. Clustering performance metrics (cluster purity, ARI, NMI) and external cross‑validation using WoS journal categories further support that OpenAlex Level‑0 labels are semantically consistent and reproducible. For convergent validity, the SBERT‑based semantic interdisciplinarity indicator showed moderate but highly significant positive correlations with the two conventional metrics (r = 0.333–0.347, p < 0.001), confirming it captures shared aspects of interdisciplinarity with traditional reference-based measures. Nested regression models further indicated that the semantic indicator provided a small but meaningful incremental gain in explanatory power (Δ adjusted R² = 0.003) after controlling for traditional indices, while its coefficient changed from positive and statistically significant in the baseline model (β = 0.5564, p < 0.01, Table 8) to marginally significant and negative in the augmented model (β = −1.5565, p = 0.085). This sign reversal reflects their complementary nature: traditional indices capture the disciplinary breadth of references, while SBERT captures semantic divergence from the home discipline; together they improve prediction accuracy, confirming our SBERT‑based measure captures unique, content‑driven interdisciplinary information beyond reference-based indicators.

Our baseline regressions confirm a robust positive association between the SBERT-based interdisciplinarity score and citation impact, consistent across OLS (β = 0.556, p < 0.01) and negative binomial specifications (β = 1.441, p < 0.01). This aligns with foundational work documenting a citation premium for interdisciplinary research [4,19,20], but contrasts with studies reporting null or negative effects [6]. We attribute this longstanding discrepancy largely to measurement differences: traditional reference-based diversity indicators capture the breadth of disciplinary inputs but not genuine semantic integration of cross-disciplinary knowledge [21], while our embedding-based approach directly quantifies a paper’s conceptual proximity to other fields, may offer a more valid measure of substantive interdisciplinarity.

Beyond average effects, our domain-level decomposition analyses—based on three data-driven groups identified via hierarchical clustering of disciplinary semantic vectors—reveal a notable asymmetric pattern: the citation premium of interdisciplinarity appears to be driven primarily by cross-domain integration whereas within-domain similarity shows no significant beneficial effects and in some cases is negatively associated with impact. This aligns with core cognitive distance theory, which posits that the greatest innovative potential may come from integrating epistemically distant fields [22], and the concept of “distal interdisciplinarity” [6,9]. By contrast, within-domain combinations may be perceived as incremental and redundant, offering limited novelty relative to disciplinary norms. Our pairwise and discipline-specific analyses further underscore this pattern: the returns to interdisciplinarity are highly contingent on specific disciplinary pairings [4], with fields like philosophy and geography acting as consistent cross-domain bridges [23,24], while many within-domain combinations deliver no impact benefits. For example, biology papers benefit from similarity to computer science and political science, but not other natural sciences; art papers see gains from proximity to history, but penalties from alignment with geology.

Existing bibliometric research has established two classic patterns: disciplinary variety is positively associated with citation impact, whereas extreme disciplinary disparity follows an inverted‑U trend. Yegros‑Yegros et al. reported that broad multidisciplinary engagement improves citations, while overly disparate knowledge combinations may incur implicit penalties [6]. Wang et al. further revealed that variety and disparity benefit long‑term citations but suppress short‑term performance, with balanced disciplinary distribution showing consistently negative outcomes [4]. The present study partially diverges from such findings, which can be attributed to differences in interdisciplinary measurement approaches between reference‑based classification and semantic embedding. We also acknowledge that our sample coverage and time window cannot fully replicate the curvilinear trend observed in long‑period bibliometric research.

Scholarly attention has also been paid to the temporal lag and high variance of interdisciplinary impact. Zhang et al. and Wang et al. confirmed the delayed citation peak and short‑term under‑recognition of interdisciplinary and novel research [25,26]. Larivière et al. indicated that most interdisciplinary co‑cited pairs form mutually beneficial relationships, while distant disciplinary links yield higher citation advantages [19]. Shi et al. noted that cross‑domain bridging patterns exist across both low‑ and high‑impact papers, reflecting high result variance [27]. Our journal‑tier heterogeneous findings offer supplementary empirical support to these views; nevertheless, the limited time span of our dataset restricts the ability to capture long‑term citation dynamics, which remains an inherent limitation of this study.

In terms of cross‑domain knowledge flow between social sciences and natural sciences, prior studies have documented obvious asymmetric characteristics. Zhou et al, Liu et al, and Chen et al. illustrated differences in cross‑disciplinary influence intensity, neighbor effect patterns, and disciplinary heterogeneity between STEM and social science fields [28–30]. The cross‑domain asymmetric effect identified in our work is generally consistent with these conclusions. Minor mismatches may arise from discrepancies in disciplinary grouping rules and sample structure, and we recognize that unbalanced sample sizes across individual disciplines may also lead to subtle empirical deviations.

We further explored heterogeneous effects across journal influence tiers and found that semantic interdisciplinarity is significantly and positively associated with citation impact in all four tiers (all p < 0.001), with the strongest coefficient observed in mid-tier journals (Tier 3). In contrast to some prior accounts of a “high-risk, high-reward” pattern concentrated in the upper tail of citation impact, our results suggest that the benefits of semantic interdisciplinarity are stable across journal tiers and most pronounced at moderate levels of journal influence, which may reflect the gradual recognition and diffusion of cross-domain research.

These findings have clear implications for research evaluation, science policy, and scholarly practice. First, our results caution against undifferentiated interdisciplinarity metrics that reward all cross-disciplinary work equally [31,32]. Evaluations and policies should prioritise not just the presence of interdisciplinarity, but the nature of disciplinary combinations, particularly through targeted cross-domain funding schemes and evaluation criteria that reward substantive knowledge integration over superficial disciplinary breadth, may be more effective at fostering high-impact research than promoting within-domain diversity, which may yield no benefits or even penalties. For individual researchers, our results show that cross-domain work appears to offer relatively strong returns, meaning early-career scholars may balance high-risk cross-domain projects with conventional work, while senior researchers are better positioned to absorb the uncertainty of distal interdisciplinarity [9]. Optimal strategies are also highly field-specific, with returns varying sharply by home discipline and target pairing.

Several limitations of this study should be acknowledged, with corresponding directions for future research. First, while our SBERT-based semantic measure improves on traditional reference-based indicators, it remains a proxy for genuine knowledge integration, and cannot distinguish between superficial cross-disciplinary mention and substantive synthesis [21]. Future work could combine semantic embeddings with citation context analysis to unpack how different modes of integration shape impact. Second, our 2015–2025 observation window cannot capture long-term citation trajectories, as prior work notes interdisciplinary research may experience delayed recognition [4]; extended follow-up periods would test the persistence of our findings. Third, our models do not account for journal prestige, author characteristics, or institutional factors that may confound the interdisciplinarity-impact relationship, and our sample is limited to OpenAlex-indexed journal articles, excluding key outputs like monographs and conference proceedings, with small samples for some humanities and engineering fields (philosophy, art, engineering). Finally, while our controls and fixed effects mitigate omitted variable bias, causal interpretation remains tentative; future research could use quasi-experimental designs to establish more robust causal evidence.

Despite these limitations, this study advances understanding of the heterogeneous relationship between interdisciplinarity and scholarly impact. Using a state-of-the-art semantic embedding approach across the full disciplinary spectrum, we help address longstanding inconsistencies in the literature, document a robust cross-domain asymmetry in the returns to interdisciplinarity, and validate that semantic interdisciplinarity delivers stable citation benefits across journal tiers. Our findings underscore the need for nuanced, context-aware approaches to interdisciplinarity in research evaluation and policy, moving beyond one-size-fits-all metrics to prioritise cross-domain integration that drives scientific breakthroughs.

## Supporting information

S1 Table Mapping correspondence between WoS fine-grained subject categories and OpenAlex Level 0 disciplines. (XLSX)
