# Supplementary material for: Beyond citation-based metrics: Measuring interdisciplinarity via SBERT semantic embeddings and its heterogeneous effects on citation impact
Source: PLoS One. 2026 Jul 22;21(7):e0354129. doi: 10.1371/journal.pone.0354129 (PMC13390828; doi:10.1371/journal.pone.0354129)
Supplement: Attachment — Submitted filename: 1.Response to reviewers.docx [file pone.0354129.s002.docx]

**Response to Comments**

**Reviewer #1:**

Thank you for your insightful, encouraging, and highly constructive comments. We greatly appreciate your recognition of the value of our work and your thoughtful suggestions for improvement. We have carefully addressed all your comments, and the specific revisions are detailed below:

**Comment 1:** "This is a well-done paper with findings that confirm many other findings in the study of citation analysis for bibliometrics... The picture that emerges is nuanced: cross-domain citation behavior is broadly a 'high risk, high reward' strategy..."

**Response:** Thank you for this accurate summary of our findings. We fully agree that our results align with and extend the existing literature on the relationship between interdisciplinarity and citation impact. To strengthen this connection, we have significantly expanded the Discussion section to explicitly compare our findings with the key studies you cited:

- We have integrated your observation about the "high risk, high reward" nature of cross-domain research and linked it to our finding that the citation premium is driven primarily by cross-domain rather than within-domain integration. Page 32-35, Lines 520-535.

- We have added detailed comparisons with Yegros-Yegros et al. (2015), Wang et al. (2015), Zhang et al. (2024), Larivière et al. (2015), and Zhou et al. (2024), highlighting both the consistencies and the unique contributions of our semantic embedding approach. Page 40-42, Lines 632-664.

We have explicitly acknowledged that our results are generally consistent with prior work but provide new insights by decomposing interdisciplinarity into within-domain and cross-domain components using content-based semantic measures rather than reference-based diversity metrics.

**Comment 2:** "The work could be made more beneficial to the community by comparing the results of their methodology with other methodologies and telling us by how much the BERT method improves upon other methods. Is this method an improvement upon other IDR methods, and if so, why and how?"

**Response:** This is an excellent suggestion that has significantly strengthened our manuscript. We have added a comprehensive "Convergent Validity and Incremental Explanatory Power" section that systematically compares our SBERT-based semantic interdisciplinarity indicator with the two most widely used classic interdisciplinarity metrics: the Simpson Diversity Index and the Rao-Stirling Index.

The key findings of this comparison are:

**1.Convergent validity:** The SBERT indicator shows moderate but highly significant positive correlations with both the Simpson Diversity Index (r = 0.333, p < 0.001) and the Rao-Stirling Index (r = 0.347, p < 0.001, Table 4a). This indicates that our semantic measure captures similar aspects of interdisciplinarity while also containing unique information not reflected in reference-based metrics. Page 26-27, Lines 419-440.

**2.Incremental explanatory power:** Nested regression models show that adding the SBERT indicator to a model already containing the two traditional metrics produces a small but statistically meaningful increase in adjusted R² (from 0.506 to 0.509, ΔR² = 0.003, Table 4b). Notably, the coefficient for the SBERT indicator changes from positive and significant in the baseline model (β = 0.5564, p < 0.01, Table 5) to negative and marginally significant in the augmented model (β = −1.5565, p = 0.085). Page 27-28, Lines 441-449.

This sign reversal is not a limitation of our measure, but rather a statistical phenomenon that reveals the complementary nature of semantic and reference-based metrics. Traditional citation-based indicators primarily capture the breadth of interdisciplinary engagement—how many different disciplines a paper cites. In contrast, the SBERT indicator captures the depth of semantic divergence from the home discipline's core knowledge. When both measures are included in the same model, they each suppress the variance in the other that is unrelated to citation impact, resulting in a more accurate overall prediction..

3. Methodological advantages: We have also clarified in the Introduction the key advantages of our semantic approach over traditional citation-based metrics: it directly measures knowledge integration at the textual level, is sensitive to cognitive distance between disciplines, and is not tied to static journal classification systems. Page 5-6, Lines 89-113.

We believe these revisions have significantly enhanced the methodological contribution of our work and demonstrate the unique value of the SBERT-based approach to measuring interdisciplinarity. **Reviewer #2:**

Thank you for your rigorous, critical, and highly valuable comments. Your concerns about methodological validity and statistical rigor have been instrumental in strengthening the quality of our manuscript. We have comprehensively addressed all your points with substantial revisions and additional analyses, as detailed below:

**Comment 1:** “First, the use of the OpenAlex concept field to assign disciplines is somewhat questionable. According to OpenAlex documentation, these concepts are generated through a hierarchical topic modeling process rather than expert curation. This introduces a level of uncertainty in how disciplines are defined and assigned. The manuscript does not sufficiently justify why these concepts can serve as a reliable proxy for disciplinary boundaries, especially given the importance of this step for the entire analysis.”

**Response:** Thank you for your critical comment. We fully agree that the disciplinary classification based on OpenAlex is the core methodological foundation of this study. We have conducted comprehensive multi-level analyses to confirm the reliability of OpenAlex disciplinary classification:

1. **UMAP semantic space visualization:** We projected the high-dimensional SBERT embeddings of all papers into a 2D space (Fig1.A). The results clearly show that the 19 disciplines form independent, well-separated clusters, with intuitively distinguishable disciplinary boundaries. Page 22, Lines 385-392.
2. **Independent-samples t-test for intra- and inter-disciplinary semantic similarity:** We calculated the cosine similarity between each paper’s embedding and its home discipline prototype vector (intra-disciplinary similarity), as well as the average similarity to the prototype vectors of the other 18 disciplines (inter-disciplinary similarity, Table 3). Welch’s t-test (unequal variances) was used for statistical testing. The results show that intra-disciplinary similarity was significantly higher than inter-disciplinary similarity for all 19 disciplines (p<0.001; Table 3), proving that the semantics within each discipline are highly concentrated and the boundaries between disciplines are clear and stable. Page 22-25, Lines 402-418.
3. **Validation by hierarchical clustering of disciplines:** We constructed a 19×19 cosine similarity matrix based on the prototype vectors of the 19 disciplines, defined the distance as 1−cosine similarity, and performed unsupervised hierarchical clustering using Ward’s method. The 19 disciplines were automatically divided into three natural clusters consistent with academic common sense: Cluster 1: Social Sciences & Humanities (political science, sociology, philosophy, art, history); Cluster 2: Applied & Interdisciplinary Sciences (geology, environmental science, geography, psychology, business, economics, chemistry); Cluster 3: Natural Sciences & Engineering (materials science, biology, medicine, physics, engineering, computer science, mathematics). The clustering results are highly consistent with traditional disciplinary classifications. We have replaced the original manual classification of disciplines with this data-driven clustering result to re-analyze intra- and inter-domain interdisciplinarity. Page 22-25, Lines 402-418.
4. **Global and discipline-specific clustering consistency tests:** We calculated three authoritative clustering indicators to quantitatively verify the clustering performance: **Clustering purity**: reflects the disciplinary purity within each cluster; **Adjusted Rand Index (ARI)**: eliminates random interference and measures the overall consistency between clustering results and true OpenAlex labels; **Normalized Mutual Information (NMI)**: measures the degree of information sharing between the two label systems. The results show a global clustering purity of 0.664, ARI=0.456, and NMI=0.634 (Table 3), further proving that the semantics of each discipline can be clearly distinguished by the SBERT embeddings. Page 22-25, Lines 402-418.
5. **External cross-validation with WoS journal classification:** Since we could not directly crawl the paper-level disciplinary classification from WoS, we used the officially publicly available Web of Science journal classification dataset (Clarivate Master Journal List) as the external gold standard. The data were obtained through the pre-organized wos-core_SCIE.csv file from a public GitHub repository (https://github.com/shirAviv/journals_categories/blob/master/wos-core_SCIE.csv). We manually mapped the 178 fine-grained disciplinary categories in the WoS database to the 19 high-level OpenAlex disciplines used in this study, and the complete mapping rules are compiled in Supplementary Table 1. Finally, 69,878 valid matched samples were obtained, with an overall classification consistency rate of 35.89% (Table 3) and a consistency rate of over 70% in the natural sciences. This further confirms that OpenAlex disciplinary classification has reliable external validity from an authoritative external classification system. Page 22-25, Lines 402-418.

**Comment 2:** “Second, although the paper proposes an indicator intended to measure interdisciplinarity, there is no validation provided for this measure. The authors appear to assume that the indicator is effective, but no quantitative or qualitative evaluation is conducted to support this assumption. In its current form, the indicator is essentially based on cosine similarity from Sentence-BERT embeddings. While this may capture semantic similarity, it is not clear whether it truly reflects interdisciplinarity. Additionally, the manuscript should clearly distinguish between Sentence-BERT and the original BERT model and use the correct terminology consistently.”

**Response:** Thank you for this precise comment. The analytical framework adopted to verify the reliability of OpenAlex disciplinary classification also simultaneously enables comprehensive validation of our SBERT-based interdisciplinarity indicator, and we have standardized relevant professional terminology throughout the manuscript:

The intra- and inter-disciplinary semantic similarity results demonstrate that semantic features extracted by SBERT can effectively distinguish the content of different academic disciplines, verifying its capability to identify intrinsic disciplinary differences. Meanwhile, the hierarchical clustering outcomes further prove that SBERT embeddings can accurately reflect the semantic proximity and distance between various disciplines. On this basis, our interdisciplinarity indicator calculated from SBERT semantic similarity is validated to appropriately and reliably reflect the actual interdisciplinary level of academic papers. In addition, we have uniformly adopted the standard term Sentence-BERT (SBERT) throughout the manuscript and explicitly distinguished it from the original BERT model to unify academic expression. Page 22-25, Lines 384-418.

**Comment 3:** “Third, the study relies on correlation analysis to examine the relationship between the proposed indicator and citation impact. However, for such analysis to be meaningful, the variables involved should be reasonably stable and well-validated. Given the concerns above regarding both the discipline classification and the interdisciplinarity measure, the resulting correlations are not entirely convincing and should be interpreted with caution.”

**Response:** Thank you for your rigorous comment. Based on the above validation procedures, the reliability of both OpenAlex disciplinary classification and SBERT semantic representation has been well confirmed. Meanwhile, To rigorously examine the relationship between semantic interdisciplinarity and citation impact, we have significantly strengthened our statistical framework with three key improvements:

**Dual model specification:** We employed both ordinary least squares (OLS) with log-transformed citation counts and negative binomial regression with raw citation counts to account for the severe overdispersion characteristic of citation data. Page 29-30, Lines 470-493.

**Comprehensive controls:** All models include paper-level confounding variables (number of authors, number of references, open access status), as well as discipline fixed effects and year fixed effects to absorb unobserved heterogeneity across fields and time periods. Page 29-30, Lines 470-493.

**Cluster-robust standard errors:** Standard errors are clustered at the discipline level to account for intra-disciplinary correlation in citation patterns. Page 31-32, Lines 501-508.

To address the critical reverse causality endogeneity problem that arises when using citation counts to both stratify journals and measure research impact, we abandoned the original citation-count-based journal stratification approach and instead adopted a more rigorous discipline-specific stratification framework using the 2025 SCImago Journal Rank (SJR) indicator; notably, we constructed journal tiers independently within each discipline rather than using cross-disciplinary quartiles to account for systematic differences in citation practices across fields, merging SJR values to individual papers at the journal level (achieving a matching rate of 89.3%), then ranking all journals within each discipline by their SJR scores and dividing them into four equal-sized tiers (Tier 1 = top 25% most influential journals in the discipline, Tier 4 = bottom 25%), a revision that effectively eliminates the endogeneity bias introduced by circularly using citation counts for both journal ranking and impact measurement and significantly improves the rationality and robustness of our heterogeneous effect analysis. Page 36-37, Lines 557-573.

Furthermore, throughout the manuscript, we adopt prudent and objective language when interpreting empirical associations. All conclusions are strictly drawn based on regression coefficients and statistical significance. We avoid overinterpretation and ensure that all inferences are rigorous and restrained.

We hope these revisions have significantly enhanced the rigor of our statistical analysis and ensured that our conclusions are appropriately tempered by the limitations of the study design.
