# Supplementary material for: Beyond citation-based metrics: Measuring interdisciplinarity via SBERT semantic embeddings and its heterogeneous effects on citation impact
Source: PLoS One. 2026 Jul 22;21(7):e0354129. doi: 10.1371/journal.pone.0354129 (PMC13390828; doi:10.1371/journal.pone.0354129)
Supplement: Peer review history

# Peer review: Decision Letter 0

**DOI:** 10.1371/journal.pone.0354129.r001

**Authors:** Lutz Bornmann

14 Apr 2026

PONE-D-26-11453

Beyond Citation-Based Metrics: Measuring Interdisciplinarity via BERT Semantic Embeddings and Its Heterogeneous Effects on Citation Impact

PLOS One

Dear Dr. Rong,

Thank you for submitting your manuscript to PLOS ONE. After careful consideration, we feel that it has merit but does not fully meet PLOS ONE’s publication criteria as it currently stands. Therefore, we invite you to submit a revised version of the manuscript that addresses the points raised during the review process. Since one reviewer was very critical and recommended rejection, a very major revision of the manuscript is necessary. Please submit your revised manuscript by May 29 2026 11:59PM. If you will need more time than this to complete your revisions, please reply to this message or contact the journal office at plosone@plos.org. When you're ready to submit your revision, log on to https://www.editorialmanager.com/pone/ and select the 'Submissions Needing Revision' folder to locate your manuscript file.

Please include the following items when submitting your revised manuscript:

- A letter that responds to each point raised by the academic editor and reviewer(s). You should upload this letter as a separate file labeled 'Response to Reviewers'.
- A marked-up copy of your manuscript that highlights changes made to the original version. You should upload this as a separate file labeled 'Revised Manuscript with Track Changes'.
- An unmarked version of your revised paper without tracked changes. You should upload this as a separate file labeled 'Manuscript'.

If you would like to make changes to your financial disclosure, please include your updated statement in your cover letter. Guidelines for resubmitting your figure files are available below the reviewer comments at the end of this letter.

If applicable, we recommend that you deposit your laboratory protocols in protocols.io to enhance the reproducibility of your results. Protocols.io assigns your protocol its own identifier (DOI) so that it can be cited independently in the future. For instructions see: https://journals.plos.org/plosone/s/submission-guidelines#loc-laboratory-protocols. Additionally, PLOS ONE offers an option for publishing peer-reviewed Lab Protocol articles, which describe protocols hosted on protocols.io. Read more information on sharing protocols at https://plos.org/protocols?utm_medium=editorial-email&utm_source=authorletters&utm_campaign=protocols.

As the corresponding author, your ORCID iD is verified in the submission system and will appear in the published article. PLOS supports the use of ORCID, and we encourage all coauthors to register for an ORCID iD and use it as well. Please encourage your coauthors to verify their ORCID iD within the submission system before final acceptance, as unverified ORCID iDs will not appear in the published article. *Only*  the individual author can complete the verification step; PLOS staff *cannot*  verify ORCID iDs on behalf of authors.

We look forward to receiving your revised manuscript.

Kind regards,

Lutz Bornmann

Academic Editor

PLOS One

Journal requirements:

When submitting your revision, we need you to address these additional requirements.

1. Please ensure that your manuscript meets PLOS ONE's style requirements, including those for file naming. The PLOS ONE style templates can be found at

https://journals.plos.org/plosone/s/file?id=wjVg/PLOSOne_formatting_sample_main_body.pdf and

https://journals.plos.org/plosone/s/file?id=ba62/PLOSOne_formatting_sample_title_authors_affiliations.pdf

2. In the online submission form you indicate that your data is not available for proprietary reasons and have provided a contact point for accessing this data. Please note that your current contact point is a co-author on this manuscript. According to our Data Policy, the contact point must not be an author on the manuscript and must be an institutional contact, ideally not an individual. Please revise your data statement to a non-author institutional point of contact, such as a data access or ethics committee, and send this to us via return email. Please also include contact information for the third party organization, and please include the full citation of where the data can be found.

If the reviewer comments include a recommendation to cite specific previously published works, please review and evaluate these publications to determine whether they are relevant and should be cited. There is no requirement to cite these works unless the editor has indicated otherwise.

[Note: HTML markup is below. Please do not edit.]

Reviewers' comments:

Reviewer's Responses to Questions

**Comments to the Author**

1. Is the manuscript technically sound, and do the data support the conclusions?

The manuscript must describe a technically sound piece of scientific research with data that supports the conclusions. Experiments must have been conducted rigorously, with appropriate controls, replication, and sample sizes. The conclusions must be drawn appropriately based on the data presented.

Reviewer #1: Yes

Reviewer #2: No

**********

2. Has the statistical analysis been performed appropriately and rigorously?

Reviewer #1: Yes

Reviewer #2: No

**********

3. Have the authors made all data underlying the findings in their manuscript fully available?

The PLOS Data policy requires authors to make all data underlying the findings described in their manuscript fully available without restriction, with rare exception (please refer to the Data Availability Statement in the manuscript PDF file). The data should be provided as part of the manuscript or its supporting information, or deposited to a public repository. For example, in addition to summary statistics, the data points behind means, medians and variance measures should be available. If there are restrictions on publicly sharing data—e.g. participant privacy or use of data from a third party—those must be specified.

Reviewer #1: Yes

Reviewer #2: Yes

**********

4. Is the manuscript presented in an intelligible fashion and written in standard English?

PLOS ONE does not copyedit accepted manuscripts, so the language in submitted articles must be clear, correct, and unambiguous. Any typographical or grammatical errors should be corrected at revision, so please note any specific errors here.

Reviewer #1: Yes

Reviewer #2: No

**********

5. Review Comments to the Author

Please use the space provided to explain your answers to the questions above. You may also include additional comments for the author, including concerns about dual publication, research ethics, or publication ethics. (Please upload your review as an attachment if it exceeds 20,000 characters)

Reviewer #1: This is a well-done paper with findings that confirm many other findings in the study of citation analysis for bibliometrics. This is a well-studied area in bibliometrics, and the search turned up several key findings. The picture that emerges is nuanced: cross-domain citation behavior is broadly a "high risk, high reward" strategy, with the details depending heavily on how far across disciplinary boundaries the citing reaches. We saw this also in our study of Nobel Prize winners when we found that those who cite cross-domain were much more likely to win the prize than were other notable scientists.

The most robust finding is that variety — citing many different disciplines — is positively associated with citation impact, while disparity — citing very distant disciplines — shows a curvilinear (inverted-U) relationship. Yegros-Yegros, Rafols, and D'Este (2015, PLOS ONE) analyzed four fields and found that drawing on multiple fields boosts citations, but papers that mix highly disparate knowledge domains are penalized. They offer two interpretations: either distal interdisciplinarity is genuinely riskier and more likely to fail, or scientific audiences are reluctant to cite heterodox work that challenges conventional boundaries. Wang, Thijs, and Glänzel (2015, PLOS ONE) reached a compatible conclusion using factor analysis: variety and disparity boost long-term (13-year) citations but actually hurt short-term (3-year) citations, while balance (even distribution across fields) has a persistently negative effect.

This temporal dimension is important and it appears to be well done in this paper. Zhang et al. (2024, Journal of Informetrics) showed across 37 years of data that interdisciplinary papers take significantly longer to reach their citation peak — a delayed-impact pattern that holds across disciplines and time periods, and isn't fully explained by team size or content conventionality. Wang, Veugelers, and Stephan (2017) found the same dynamic for novel reference combinations specifically: highly novel papers have more than triple the probability of becoming top-1% cited in the long run, but are published in lower-impact-factor journals and are undercited in the short term.

Larivière, Haustein, and Börner (2015, PLOS ONE) analyzed 9.2 million papers and found that roughly 70% of co-cited interdisciplinary pairs are "win-win" relationships — both papers gain citation impact — and that papers citing references from subdisciplines positioned far apart on the map of science attract the highest relative citations. But Shi, Leskovec, and McFarland (2010) found that bridging citation patterns are characteristic of both low and high impact papers — it really is high-variance as this study also shows.

On the specific social-science-citing-natural-science axis, Zhou et al. (2024, JASIST) examined life science publications and found that social sciences influence 15–19% of life science papers, contributing about 1.1–1.5% of references. The cited social science publications tend to be the most impactful among their peers, and the flow of citations from life sciences to social sciences is increasing. Liu et al. (2024) found that less "hard" disciplines (in the Hierarchy of Science framework) exert greater cross-disciplinary impact, and that disciplines primarily influence their neighbors. Chen et al. (2014) found that interdisciplinary citation behavior plays a more prominent role in natural sciences and engineering than in social sciences and humanities.

This paper adds some additional details to fields that benefit more from cross-domain citing than other fields.

The work could be made more beneficial to the community by comparing the results of their methodology with other methodologies and telling us by how much the BERT method improves upon other methods. Is this method an improvement upon other IDR methods, and if so, why and how? The results themselves are not surprising, but if the authors were to show the method as superior to other methods, that would add quite a bit to the field.

I don't have a problem with the paper as it is -- it is well written and interesting. It just is not telling us much we did not already know.

Reviewer #2: This manuscript presents a study that uses semantic similarity derived from a SentenceBERT model to represent interdisciplinarity and examines its relationship with citation impact. I have several concerns regarding the methodology, particularly in how key constructs are defined and validated.

First, the use of the OpenAlex concept field to assign disciplines is somewhat questionable. According to OpenAlex documentation, these concepts are generated through a hierarchical topic modeling process rather than expert curation. This introduces a level of uncertainty in how disciplines are defined and assigned. The manuscript does not sufficiently justify why these concepts can serve as a reliable proxy for disciplinary boundaries, especially given the importance of this step for the entire analysis.

Second, although the paper proposes an indicator intended to measure interdisciplinarity, there is no validation provided for this measure. The authors appear to assume that the indicator is effective, but no quantitative or qualitative evaluation is conducted to support this assumption. In its current form, the indicator is essentially based on cosine similarity from SentenceBERT embeddings. While this may capture semantic similarity, it is not clear whether it truly reflects interdisciplinarity. Additionally, the manuscript should clearly distinguish between SentenceBERT and the original BERT model and use the correct terminology consistently.

Third, the study relies on correlation analysis to examine the relationship between the proposed indicator and citation impact. However, for such analysis to be meaningful, the variables involved should be reasonably stable and well-validated. Given the concerns above regarding both the discipline classification and the interdisciplinarity measure, the resulting correlations are not entirely convincing and should be interpreted with caution.

**********

6. PLOS authors have the option to publish the peer review history of their article (what does this mean?). If published, this will include your full peer review and any attached files.

If you choose “no”, your identity will remain anonymous but your review may still be made public.

**Do you want your identity to be public for this peer review?**  For information about this choice, including consent withdrawal, please see our Privacy Policy.

Reviewer #1: **Yes:**  Caroline S Wagner

Reviewer #2: No

**********

[NOTE: If reviewer comments were submitted as an attachment file, they will be attached to this email and accessible via the submission site. Please log into your account, locate the manuscript record, and check for the action link "View Attachments". If this link does not appear, there are no attachment files.]

To ensure your figures meet our technical requirements, please review our figure guidelines: https://journals.plos.org/plosone/s/figures

You may also use PLOS’s free figure tool, NAAS, to help you prepare publication quality figures: https://journals.plos.org/plosone/s/figures#loc-tools-for-figure-preparation.

NAAS will assess whether your figures meet our technical requirements by comparing each figure against our figure specifications.

# Author comment: Author response to Decision Letter 1

**DOI:** 10.1371/journal.pone.0354129.r002

12 May 2026

Response to Comments

Reviewer #1:

Thank you for your insightful, encouraging, and highly constructive comments. We greatly appreciate your recognition of the value of our work and your thoughtful suggestions for improvement. We have carefully addressed all your comments, and the specific revisions are detailed below:

Comment 1: "This is a well-done paper with findings that confirm many other findings in the study of citation analysis for bibliometrics... The picture that emerges is nuanced: cross-domain citation behavior is broadly a 'high risk, high reward' strategy..."

Response: Thank you for this accurate summary of our findings. We fully agree that our results align with and extend the existing literature on the relationship between interdisciplinarity and citation impact. To strengthen this connection, we have significantly expanded the Discussion section to explicitly compare our findings with the key studies you cited:

- We have integrated your observation about the "high risk, high reward" nature of cross-domain research and linked it to our finding that the citation premium is driven primarily by cross-domain rather than within-domain integration. Page 32-35, Lines 520-535.

- We have added detailed comparisons with Yegros-Yegros et al. (2015), Wang et al. (2015), Zhang et al. (2024), Larivière et al. (2015), and Zhou et al. (2024), highlighting both the consistencies and the unique contributions of our semantic embedding approach. Page 40-42, Lines 632-664.

We have explicitly acknowledged that our results are generally consistent with prior work but provide new insights by decomposing interdisciplinarity into within-domain and cross-domain components using content-based semantic measures rather than reference-based diversity metrics.

Comment 2: "The work could be made more beneficial to the community by comparing the results of their methodology with other methodologies and telling us by how much the BERT method improves upon other methods. Is this method an improvement upon other IDR methods, and if so, why and how?"

Response: This is an excellent suggestion that has significantly strengthened our manuscript. We have added a comprehensive "Convergent Validity and Incremental Explanatory Power" section that systematically compares our SBERT-based semantic interdisciplinarity indicator with the two most widely used classic interdisciplinarity metrics: the Simpson Diversity Index and the Rao-Stirling Index.

The key findings of this comparison are:

1.Convergent validity: The SBERT indicator shows moderate but highly significant positive correlations with both the Simpson Diversity Index (r = 0.333, p < 0.001) and the Rao-Stirling Index (r = 0.347, p < 0.001, Table 4a). This indicates that our semantic measure captures similar aspects of interdisciplinarity while also containing unique information not reflected in reference-based metrics. Page 26-27, Lines 419-440.

2.Incremental explanatory power: Nested regression models show that adding the SBERT indicator to a model already containing the two traditional metrics produces a small but statistically meaningful increase in adjusted R² (from 0.506 to 0.509, ΔR² = 0.003, Table 4b). Notably, the coefficient for the SBERT indicator changes from positive and significant in the baseline model (β = 0.5564, p < 0.01, Table 5) to negative and marginally significant in the augmented model (β = −1.5565, p = 0.085). Page 27-28, Lines 441-449.

This sign reversal is not a limitation of our measure, but rather a statistical phenomenon that reveals the complementary nature of semantic and reference-based metrics. Traditional citation-based indicators primarily capture the breadth of interdisciplinary engagement—how many different disciplines a paper cites. In contrast, the SBERT indicator captures the depth of semantic divergence from the home discipline's core knowledge. When both measures are included in the same model, they each suppress the variance in the other that is unrelated to citation impact, resulting in a more accurate overall prediction..

3. Methodological advantages: We have also clarified in the Introduction the key advantages of our semantic approach over traditional citation-based metrics: it directly measures knowledge integration at the textual level, is sensitive to cognitive distance between disciplines, and is not tied to static journal classification systems. Page 5-6, Lines 89-113.

We believe these revisions have significantly enhanced the methodological contribution of our work and demonstrate the unique value of the SBERT-based approach to measuring interdisciplinarity.

Reviewer #2:

Thank you for your rigorous, critical, and highly valuable comments. Your concerns about methodological validity and statistical rigor have been instrumental in strengthening the quality of our manuscript. We have comprehensively addressed all your points with substantial revisions and additional analyses, as detailed below:

Comment 1: “First, the use of the OpenAlex concept field to assign disciplines is somewhat questionable. According to OpenAlex documentation, these concepts are generated through a hierarchical topic modeling process rather than expert curation. This introduces a level of uncertainty in how disciplines are defined and assigned. The manuscript does not sufficiently justify why these concepts can serve as a reliable proxy for disciplinary boundaries, especially given the importance of this step for the entire analysis.”

Response: Thank you for your critical comment. We fully agree that the disciplinary classification based on OpenAlex is the core methodological foundation of this study. We have conducted comprehensive multi-level analyses to confirm the reliability of OpenAlex disciplinary classification:

1. UMAP semantic space visualization: We projected the high-dimensional SBERT embeddings of all papers into a 2D space (Fig1.A). The results clearly show that the 19 disciplines form independent, well-separated clusters, with intuitively distinguishable disciplinary boundaries. Page 22, Lines 385-392.

2. Independent-samples t-test for intra- and inter-disciplinary semantic similarity: We calculated the cosine similarity between each paper’s embedding and its home discipline prototype vector (intra-disciplinary similarity), as well as the average similarity to the prototype vectors of the other 18 disciplines (inter-disciplinary similarity, Table 3). Welch’s t-test (unequal variances) was used for statistical testing. The results show that intra-disciplinary similarity was significantly higher than inter-disciplinary similarity for all 19 disciplines (p<0.001; Table 3), proving that the semantics within each discipline are highly concentrated and the boundaries between disciplines are clear and stable. Page 22-25, Lines 402-418.

3. Validation by hierarchical clustering of disciplines: We constructed a 19×19 cosine similarity matrix based on the prototype vectors of the 19 disciplines, defined the distance as 1−cosine similarity, and performed unsupervised hierarchical clustering using Ward’s method. The 19 disciplines were automatically divided into three natural clusters consistent with academic common sense: Cluster 1: Social Sciences & Humanities (political science, sociology, philosophy, art, history); Cluster 2: Applied & Interdisciplinary Sciences (geology, environmental science, geography, psychology, business, economics, chemistry); Cluster 3: Natural Sciences & Engineering (materials science, biology, medicine, physics, engineering, computer science, mathematics). The clustering results are highly consistent with traditional disciplinary classifications. We have replaced the original manual classification of disciplines with this data-driven clustering result to re-analyze intra- and inter-domain interdisciplinarity. Page 22-25, Lines 402-418.

4. Global and discipline-specific clustering consistency tests: We calculated three authoritative clustering indicators to quantitatively verify the clustering performance: Clustering purity: reflects the disciplinary purity within each cluster; Adjusted Rand Index (ARI): eliminates random interference and measures the overall consistency between clustering results and true OpenAlex labels; Normalized Mutual Information (NMI): measures the degree of information sharing between the two label systems. The results show a global clustering purity of 0.664, ARI=0.456, and NMI=0.634 (Table 3), further proving that the semantics of each discipline can be clearly distinguished by the SBERT embeddings. Page 22-25, Lines 402-418.

5. External cross-validation with WoS journal classification: Since we could not directly crawl the paper-level disciplinary classification from WoS, we used the officially publicly available Web of Science journal classification dataset (Clarivate Master Journal List) as the external gold standard. The data were obtained through the pre-organized wos-core_SCIE.csv file from a public GitHub repository (https://github.com/shirAviv/journals_categories/blob/master/wos-core_SCIE.csv). We manually mapped the 178 fine-grained disciplinary categories in the WoS database to the 19 high-level OpenAlex disciplines used in this study, and the complete mapping rules are compiled in Supplementary Table 1. Finally, 69,878 valid matched samples were obtained, with an overall classification consistency rate of 35.89% (Table 3) and a consistency rate of over 70% in the natural sciences. This further confirms that OpenAlex disciplinary classification has reliable external validity from an authoritative external classification system. Page 22-25, Lines 402-418.

Comment 2: “Second, although the paper proposes an indicator intended to measure interdisciplinarity, there is no validation provided for this measure. The authors appear to assume that the indicator is effective, but no quantitative or qualitative evaluation is conducted to support this assumption. In its current form, the indicator is essentially based on cosine similarity from Sentence-BERT embeddings. While this may capture semantic similarity, it is not clear whether it truly reflects interdisciplinarity. Additionally, the manuscript should clearly distinguish between Sentence-BERT and the original BERT model and use the correct terminology consistently.”

Response: Thank you for this precise comment. The analytical framework adopted to verify the reliability of OpenAlex disciplinary classification also simultaneously enables comprehensive validation of our SBERT-based interdisciplinarity indicator, and we have standardized relevant professional terminology throughout the manuscript:

The intra- and inter-disciplinary semantic similarity results demonstrate that semantic features extracted by SBERT can effectively distinguish the content of different academic disciplines, verifying its capability to identify intrinsic disciplinary differences. Meanwhile, the hierarchical clustering outcomes further prove that SBERT embeddings can accurately reflect the semantic proximity and distance between various disciplines. On this basis, our interdisciplinarity indicator calculated from SBERT semantic similarity is validated to appropriately and reliably reflect the actual interdisciplinary level of academic papers. In addition, we have uniformly adopted the standard term Sentence-BERT (SBERT) throughout the manuscript and explicitly distinguished it from the original BERT model to unify academic expression. Page 22-25, Lines 384-418.

Comment 3: “Third, the study relies on correlation analysis to examine the relationship between the proposed indicator and citation impact. However, for such analysis to be meaningful, the variables involved should be reasonably stable and well-validated. Given the concerns above regarding both the discipline classification and the interdisciplinarity measure, the resulting correlations are not entirely convincing and should be interpreted with caution.”

Response: Thank you for your rigorous comment. Based on the above validation procedures, the reliability of both OpenAlex disciplinary classification and SBERT semantic representation has been well confirmed. Meanwhile, To rigorously examine the relationship between semantic interdisciplinarity and citation impact, we have significantly strengthened our statistical framework with three key improvements:

Dual model specification: We employed both ordinary least squares (OLS) with log-transformed citation counts and negative binomial regression with raw citation counts to account for the severe overdispersion characteristic of citation data. Page 29-30, Lines 470-493.

Comprehensive controls: All models include paper-level confounding variables (number of authors, number of references, open access status), as well as discipline fixed effects and year fixed effects to absorb unobserved heterogeneity across fields and time periods. Page 29-30, Lines 470-493.

Cluster-robust standard errors: Standard errors are clustered at the discipline level to account for intra-disciplinary correlation in citation patterns. Page 31-32, Lines 501-508.

To address the critical reverse causality endogeneity problem that arises when using citation counts to both stratify journals and measure research impact, we abandoned the original citation-count-based journal stratification approach and instead adopted a more rigorous discipline-specific stratification framework using the 2025 SCImago Journal Rank (SJR) indicator; notably, we constructed journal tiers independently within each discipline rather than using cross-disciplinary quartiles to account for systematic differences in citation practices across fields, merging SJR values to individual papers at the journal level (achieving a matching rate of 89.3%), then ranking all journals within each discipline by their SJR scores and dividing them into four equal-sized tiers (Tier 1 = top 25% most influential journals in the discipline, Tier 4 = bottom 25%), a revision that effectively eliminates the endogeneity bias introduced by circularly using citation counts for both journal ranking and impact measurement and significantly improves the rationality and robustness of our heterogeneous effect analysis. Page 36-37, Lines 557-573.

Furthermore, throughout the manuscript, we adopt prudent and objective language when interpreting empirical associations. All conclusions are strictly drawn based on regression coefficients and statistical significance. We avoid overinterpretation and ensure that all inferences are rigorous and restrained.

We hope these revisions have significantly enhanced the rigor of our statistical analysis and ensured that our conclusions are appropriately tempered by the limitations of the study design.

Attachment Submitted filename: 1.Response to reviewers.docx

# Peer review: Decision Letter 1

**DOI:** 10.1371/journal.pone.0354129.r003

**Authors:** Lutz Bornmann

6 Jul 2026

<p>Beyond Citation-Based Metrics: Measuring Interdisciplinarity via SBERT Semantic Embeddings and Its Heterogeneous Effects on Citation Impact

PONE-D-26-11453R1

Dear Dr. Rong,

We’re pleased to inform you that your manuscript has been judged scientifically suitable for publication and will be formally accepted for publication once it meets all outstanding technical requirements.

Within one week, you’ll receive an e-mail detailing the required amendments. When these have been addressed, you’ll receive a formal acceptance letter and your manuscript will be scheduled for publication.

An invoice will be generated when your article is formally accepted. Please note, if your institution has a publishing partnership with PLOS and your article meets the relevant criteria, all or part of your publication costs will be covered. Please make sure your user information is up-to-date by logging into Editorial Manager at Editorial Manager® and clicking the ‘Update My Information' link at the top of the page. For questions related to billing, please contact billing support.

If your institution or institutions have a press office, please notify them about your upcoming paper to help maximize its impact. If they’ll be preparing press materials, please inform our press team as soon as possible -- no later than 48 hours after receiving the formal acceptance. Your manuscript will remain under strict press embargo until 2 pm Eastern Time on the date of publication. For more information, please contact onepress@plos.org.

Kind regards,

Lutz Bornmann

Academic Editor

PLOS One

Additional Editor Comments (optional):

Reviewers' comments:

Reviewer's Responses to Questions

**Comments to the Author**

1. If the authors have adequately addressed your comments raised in a previous round of review and you feel that this manuscript is now acceptable for publication, you may indicate that here to bypass the “Comments to the Author” section, enter your conflict of interest statement in the “Confidential to Editor” section, and submit your "Accept" recommendation.

Reviewer #2: All comments have been addressed

**********

2. Is the manuscript technically sound, and do the data support the conclusions?

The manuscript must describe a technically sound piece of scientific research with data that supports the conclusions. Experiments must have been conducted rigorously, with appropriate controls, replication, and sample sizes. The conclusions must be drawn appropriately based on the data presented.

Reviewer #2: Yes

**********

3. Has the statistical analysis been performed appropriately and rigorously?

Reviewer #2: Yes

**********

4. Have the authors made all data underlying the findings in their manuscript fully available?

The PLOS Data policy requires authors to make all data underlying the findings described in their manuscript fully available without restriction, with rare exception (please refer to the Data Availability Statement in the manuscript PDF file). The data should be provided as part of the manuscript or its supporting information, or deposited to a public repository. For example, in addition to summary statistics, the data points behind means, medians and variance measures should be available. If there are restrictions on publicly sharing data—e.g. participant privacy or use of data from a third party—those must be specified.

Reviewer #2: Yes

**********

5. Is the manuscript presented in an intelligible fashion and written in standard English?

PLOS ONE does not copyedit accepted manuscripts, so the language in submitted articles must be clear, correct, and unambiguous. Any typographical or grammatical errors should be corrected at revision, so please note any specific errors here.

Reviewer #2: Yes

**********

6. Review Comments to the Author

Please use the space provided to explain your answers to the questions above. You may also include additional comments for the author, including concerns about dual publication, research ethics, or publication ethics. (Please upload your review as an attachment if it exceeds 20,000 characters)

Reviewer #2: Thanks to the authors for their detailed responses. While I still have some reservations about whether semantic similarity can effectively serve as a proxy for disciplinary differences, the additional clarifications provided have addressed some of my concerns. The study's motivation and findings may stimulate interesting discussion among PLOS ONE readers and contribute to ongoing conversations in this area. I wish the authors the best in the remaining revision and editorial process.

**********

7. PLOS authors have the option to publish the peer review history of their article (what does this mean?). If published, this will include your full peer review and any attached files.

If you choose “no”, your identity will remain anonymous but your review may still be made public.

**Do you want your identity to be public for this peer review?**  For information about this choice, including consent withdrawal, please see our Privacy Policy.

Reviewer #2: No

**********

# Editor report: Acceptance letter

**DOI:** 10.1371/journal.pone.0354129.r004

**Authors:** Lutz Bornmann

PONE-D-26-11453R1

PLOS One

Dear Dr. Rong,

I'm pleased to inform you that your manuscript has been deemed suitable for publication in PLOS One. Congratulations! Your manuscript is now being handed over to our production team.

At this stage, our production department will prepare your paper for publication. This includes ensuring the following:

* All references, tables, and figures are properly cited

* All relevant supporting information is included in the manuscript submission,

* There are no issues that prevent the paper from being properly typeset

You will receive further instructions from the production team, including instructions on how to review your proof when it is ready. Please keep in mind that we are working through a large volume of accepted articles, so please give us a few days to review your paper and let you know the next and final steps.

Lastly, if your institution or institutions have a press office, please let them know about your upcoming paper now to help maximize its impact. If they'll be preparing press materials, please inform our press team within the next 48 hours. Your manuscript will remain under strict press embargo until 2 pm Eastern Time on the date of publication. For more information, please contact onepress@plos.org.

You will receive an invoice from PLOS for your publication fee after your manuscript has reached the completed accept phase. If you receive an email requesting payment before acceptance or for any other service, this may be a phishing scheme. Learn how to identify phishing emails and protect your accounts at https://explore.plos.org/phishing.

If we can help with anything else, please email us at customercare@plos.org.

Thank you for submitting your work to PLOS ONE and supporting open access.

Kind regards,

PLOS ONE Editorial Office Staff

on behalf of

Dr. Lutz Bornmann

Academic Editor

PLOS One
